# Evaluation of a practical expert defined approach to patient population segmentation: a case study in Singapore

**DOI:** 10.1186/s12913-017-2736-8

**Published:** 2017-11-23

**Authors:** Lian Leng Low, Yu Heng Kwan, Nan Liu, Xuan Jing, Edwin Cheng Tee Low, Julian Thumboo

**Affiliations:** 1 Department of Family Medicine & Continuing Care, Singapore General Hospital, 20 College Road, Singapore, 169856 Singapore; 2 Family Medicine, Duke-NUS Medical School, Singapore, Singapore; 3 Singapore Heart Foundation, Singapore, Singapore; 4 Health Services Research Centre, Singapore Health Services, Duke-NUS Medical School, Singapore, 169857 Singapore; 5 SingHealth Regional Health System, Singapore Health Services, 20 College Road, Singapore, 169856 Singapore; 6 Department of Rheumatology and Immunology, Singapore General Hospital, 20 College Road, Singapore, 169856 Singapore

**Keywords:** Population segmentation, Integrated care, Chronic disease, Healthcare utilization, Patient segmentation

## Abstract

**Background:**

Segmenting the population into groups that are relatively homogeneous in healthcare characteristics or needs is crucial to facilitate integrated care and resource planning. We aimed to evaluate the feasibility of segmenting the population into discrete, non-overlapping groups using a practical expert and literature driven approach. We hypothesized that this approach is feasible utilizing the electronic health record (EHR) in SingHealth.

**Methods:**

In addition to well-defined segments of “Mostly healthy”, “Serious acute illness but curable” and “End of life” segments that are also present in the Ministry of Health Singapore framework, patients with chronic diseases were segmented into “Stable chronic disease”, “Complex chronic diseases without frequent hospital admissions”, and “Complex chronic diseases with frequent hospital admissions”. Using the electronic health record (EHR), we applied this framework to all adult patients who had a healthcare encounter in the Singapore Health Services Regional Health System in 2012. ICD-9, 10 and polyclinic codes were used to define chronic diseases with a comprehensive look-back period of 5 years. Outcomes (hospital admissions, emergency attendances, specialist outpatient clinic attendances and mortality) were analyzed for years 2012 to 2015.

**Results:**

Eight hundred twenty five thousand eight hundred seventy four patients were included in this study with the majority being healthy without chronic diseases. The most common chronic disease was hypertension. Patients with “complex chronic disease” with frequent hospital admissions segment represented 0.6% of the eligible population, but accounted for the highest hospital admissions (4.33 ± 2.12 admissions; *p* < 0.001) and emergency attendances (ED) (3.21 ± 3.16 ED visits; p < 0.001) per patient, and a high mortality rate (16%). Patients with metastatic disease accounted for the highest specialist outpatient clinic attendances (27.48 ± 23.68 visits; p < 0.001) per patient despite their relatively shorter course of illness and high one-year mortality rate (33%).

**Conclusion:**

This practical segmentation framework can potentially distinguish among groups of patients, and highlighted the high disease burden of patients with chronic diseases. Further research to validate this approach of population segmentation is needed.

**Electronic supplementary material:**

The online version of this article (doi: 10.1186/s12913-017-2736-8) contains supplementary material, which is available to authorized users.

## Background

Segmenting the population into groups that are relatively homogenous in terms of their healthcare needs or characteristics can facilitate the planning for resource allocation and the design of integrated care programs [1–3] or integrated practice units around these patient segments [4, 5]. Coupled with an in-depth understanding of the healthcare needs, demand and supply of healthcare provision for each population segment, this represents an excellent proposition to provide value-based population health care. Without clear segmentation into homogeneous needs or characteristics, care is likely to be delivered in a fragmented and episodic approach that is inefficient and unsustainable.

In existing literature, there are two well-established segmentation frameworks, namely the Johns Hopkins Adjusted Clinical Groups System [6] that uses a granular system of diagnosis code mapping as the basis for different groupings and the 3 M Clinical Risk Groups system [7] that distributes patients among 272 groups for a more detailed risk analysis. Other approaches to population segmentation include expert driven approaches where segments are decided a-priori through expert and thorough literature inputs; and data driven approaches where segmentation is done post-hoc using statistical methods in a data-driven manner. An example of a data-driven approach is to use latent class analysis, such as in Van der Laan et al’s demand-driven segmentation model [8], Liu et al’s study of the Taiwan National health Insurance survey participants [9] and Lafortune’s secondary analysis of SIPA trial [10] (French acronym for System of Integrated Care for Older Persons). In these studies, survey or trial data had captured functional and behavioral data allowing creation of segments such as cognitive, functional or physical impairments. However, the generalizability of such a segment classification to entire populations would be limited by the inclusion criteria of the original trials and indicators used for segmentation. To the best of our knowledge, these three data driven models [8–10] have not been replicated elsewhere.

Examples of published expert driven approaches include the Bridges to Health [11] and Senior Segmentation Algorithm [12]. The Bridges to Health describes a theoretical person centred segmentation framework but the model has not been applied beyond concept to population segmentation. The Senior Segmentation Algorithm developed at Kaiser Permanente proposed a four-group classification for elderly persons aged 65 years or older. In our review of both frameworks, there are several well-defined segments that are generalizable to other populations: 1) Healthy without chronic diseases; 2) End of Life; 3) Maternal and Infant health; 4) Acute illness but curable [11–13]. However, there is less consensus on the exact segmentation for chronic diseases, although there is a gradient based on number or severity of chronic diseases. For example, the Complexdex [14] algorithm that focused on segmentation of patients with chronic diseases classified nine prevalent chronic diseases into three complexity cohorts; namely Minor Chronic or “at risk” (individuals with hypertension or hyperlipidemia), Major Chronic (individuals with asthma, diabetes, chronic obstructive pulmonary disease, coronary artery disease etc) and System failure (heart failure and chronic kidney disease). Madotto et al. classified over 1 million patients into “Possibly affected by chronic disease”; “One chronic disease”; and “More than one chronic disease” [13].

Locally, the Ministry of Health Singapore proposed a consensus based segmentation framework to classify patients into five complexity cohorts; namely “Mostly healthy”, “Serious acute illness but curable”, “Stable chronic”, “Complex chronic”, and “End of life”. However, this proposed segmentation framework has not been validated or evaluated for its feasibility. Overall, there is absence of a consensus segmentation framework that is directly applicable to all contexts and this gap has prompted this work to develop a practical segmentation framework that is well defined for an entire population and potentially generalizable beyond the Singapore context.

In our study, we aimed to 1) assess the validity and feasibility of the proposed segmentation framework from the Ministry of Health Singapore to segment the patient population into distinct, non-overlapping patient segments, 2) describe the patient profile and health utilization in each segment. We hypothesized that segmenting the patient population accurately and meaningfully is feasible utilizing electronic health records data.

## Methods

### Study design, setting and population

We conducted a retrospective cross-sectional study designed to segment the year 2012 SingHealth RHS patient population into distinct, non-overlapping patient segments and describe the patient profile and health utilization in each segment. All adult patients (≥ 21 years of age) who have utilized services in the SingHealth RHS (outpatient, emergency department and inpatient) from 1 January 2012 to 31 December 2012 were included. We selected the year 2012 for segmentation as our electronic health record system has been well established and comprehensive since 2009, and it allowed us to evaluate outcomes from 2013 onwards. Patients were excluded if they were below 21 years of age. This study was approved by SingHealth Centralized Institutional Review Board (CIRB 2016/2294).

We extracted de-identified data from the electronic health records of Singhealth using the Oracle Business Intelligence and Enterprise Edition (OBIEE) Software. SingHealth has a well-established EHR system that integrates information from multiple sources including administrative data, clinical data and ancillary, called the Electronic Health Intelligence System (eHINTS) [15]. The electronic health records consolidated and analyzed patient and healthcare data that were uploaded on the web-based business intelligence software. As patients visit multiple healthcare institutions, data merging was done in OBIEE using unique identifier including national registration identities.

### Variables, data sources and healthcare utilization measures

Variables from the socio-demographic, chronic diseases and healthcare utilization categories were extracted for this study. Patient demographics (age, gender, and ethnicity) and prior healthcare utilization (hospital admissions, emergency department attendances, specialist outpatient clinic attendances) in the past 1 year were retrieved. Chronic diseases were derived by extracting the ICD-9, ICD-10 [16–18] codes and primary care codes dating back 5 years. We believe that this is most comprehensive among published literature and would account for potential lapses in diagnostic coding [17]. Healthcare utilization and mortality data in 2012 was also retrieved for this study.

### Segmentation classification

We selected our Ministry of Health segmentation framework which was based on expert opinion, and further modified it by expanding the complex chronic segment into 2 segments named “Complex chronic diseases without frequent hospital admissions”, and “Complex chronic diseases with frequent hospital admissions” based on the number of hospital admissions. We defined frequent hospital admissions as 3 or more hospital admissions in past 12 months [17, 19] and is a proxy marker for being a high cost user [20] in Singapore. Therefore, the workgroup decided on using frequent hospital admissions to stratify between patients with complex chronic diseases. We rationalize that complex chronic without frequent hospital admission could be cared for in primary care while complex chronic with frequent hospital admission is a high burden segment requiring additional integrated care teams and resources to systematically address risk factors for frequent hospital admission [2, 17]. This segmentation framework was endorsed by the population segmentation workgroup of the SingHealth RHS as the first cut to understanding the population profile in our care boundaries. The workgroup comprised experienced clinicians and administrators from the SingHealth polyclinics, Seng Kang Hospital, SingHealth Regional Health System, Singapore General Hospital and Duke-NUS Medical School. The definition and descriptive examples of the six segments are detailed in Additional file 1: Table S1. The list of chronic diseases was based on the 19 chronic diseases of the Singapore Chronic Disease Management Program (CDMP) [21], Charlson Comorbidity Index [22, 23] and Elixhauser Comorbidities [16] (Additional file 1: Table S2). A stable chronic disease is defined as a chronic disease that does not interfere with / restrict usual function or sufficient to trigger care seeking [11]. The classification of chronic diseases into stable chronic diseases or complex chronic diseases (Additional file 1: Table S2) was iteratively refined and agreed among all experts before we apply to our data analysis.

### Statistical analysis

Descriptive statistics were conducted to describe the patient characteristics for each segment and presented as mean ± standard deviation or number (%). We examined and compared patient demographics, prevalence of chronic diseases and hospital health services utilization using Chi-square tests for categorical variables and one-way ANOVA tests for continuous variables. We conducted all analyses using R version 3.2.3 (R Foundation, Vienna, Austria).

## Results

The Singapore Health Services (SingHealth) RHS, which is based in the largest public healthcare cluster in Singapore include the 1597 beds Academic Medical Centre Singapore General Hospital (SGH) campus, national specialty centres in ophthalmology, oncology, cardiovascular medicine, oral medicine, neuroscience, a tertiary women’s and children’s hospital, and nine large primary care facilities. Our population database included 825,874 adult patients in Singapore who were categorized into “Mostly healthy”, “Serious acute illness but curable”, “Stable chronic”, “Complex chronic without frequent hospital admissions”, “Complex chronic with frequent hospital admissions”, and “End of life” (Table 1). The mean age in our sample was 55.6 years, as compared to the median age of the Singapore population of 38.4 years. The proportion of male gender and ethnic groups in our study are similar to the Singapore demographic profile [24]. Patients with chronic diseases are similar in age but are older than patients who are in the mostly healthy and serious acute illness segments. Patients in the complex chronic with frequent hospital admission segment were more likely to be older and had higher hospital admissions and ED visits per patient in the past year compared to patients from other segments. The End of Life segment accounted for the highest specialist clinic visits per patient in the past year, reflecting the high care burden associated with the metastatic diseases.

**Table 1:** Demographics and Past Healthcare Utilization of patients in the SingHealth Regional Health System

Variable	All		Mostly Healthy		Serious Acute Illness but Curable		Stable Chronic		Complex Chronic without frequent hospital admissions		Complex chronic with frequent hospital admissions		End of Life		*p*-value
Number of patients (%)	825,874	(100)	506,101	(61.3)	44,023	(5.3)	177,550	(21.5)	90,540	(11.0)	4705	(0.6)	2955	(0.4)	<0.001
Patient Demographics															
Age (in years), mean, (SD)	55.6	(9.8)	44.4	(15.6)	42.0	(15.8)	61.6	(13.6)	61.1	(15.1)	64.6	(14.5)	60.0	(13.0)	<0.001
21 – 39 (%)	261,095	(32)	217,648	(43)	23,898	(54)	10,873	(6)	8217	(9)	279	(6)	180	(6)	
40 – 64 (%)	384,596	(47)	230,499	(46)	15,011	(34)	92,032	(52)	43,387	(48)	1955	(42)	1712	(58)	
65 – 84 (%)	166,354	(20)	54,890	(11)	4646	(11)	68,607	(39)	35,063	(39)	2157	(46)	991	(34)	
≥ 85 (%)	13,829	(2)	3064	(1)	468	(1)	6038	(3)	3873	(4)	314	(7)	72	(2)	
Gender															
Male (%)	347,704	(42)	209,969	(41)	12,620	(29)	79,196	(45)	42,186	(47)	2447	(52)	1286	(44)	<0.001
Ethnicity															
Chinese (%)	584,289	(71)	346,461	(68)	25,617	(58)	137,299	(77)	69,173	(76)	3415	(73)	2324	(79)	<0.001
Indian (%)	70,657	(9)	45,406	(9)	5121	(12)	12,286	(7)	7248	(8)	469	(10)	127	(4)	
Malay (%)	88,842	(11)	50,589	(10)	7022	(16)	21,226	(12)	9152	(10)	614	(13)	239	(8)	
Others (%)	82,086	(10)	63,645	(13)	6263	(14)	6739	(4)	4967	(5)	207	(4)	265	(9)	
Past Healthcare Utilization per patient in the past year (2011)															
ED visits, total (%)	0.11		0.05		0.10		0.10		0.34		1.55		0.45		<0.001
Specialist Clinic visits, total (%)	2.78		1.87		3.11		2.28		7.61		13.6		20.05		<0.001
Hospital admissions, total (%)	0.10		0.04		0.12		0.07		0.36		1.71		0.77		<0.001

Abbreviations: Emergency Department (ED). Numbers were presented as mean ± standard deviation or number (%) as appropriate

Healthcare utilization and mortality differed among the population segments (Table 2). Patients from the “complex chronic with frequent hospital admissions” segment had a disproportionate healthcare utilization in the form of hospital admissions, ED visits, while the “End of life” segment accounted for the highest specialist clinic visits despite the high mortality rate in this segment. Table 2 presented the healthcare utilization per patient in 2012 for emergency department (ED) visits, specialist clinic visits and hospital admissions, and mortality rate in 2012. The “complex chronic diseases with frequent hospital admissions” patient segment had the highest healthcare utilization for emergency department (ED) (3.21 ± 3.16 ED visits; *p* < 0.001) and hospital admissions (4.33 ± 2.12 hospital admissions per patient; *p* < 0.001). In addition, the one-year mortality rate of “Complex chronic disease with frequent hospital admissions” patients was 16%, significantly higher than the mortality rates of other patients excluding “End of life” patients. The “End of life” patient segment had the highest mortality rate 33% and specialist clinic visits (27.48 ± 23.68 visits; *p* < 0.001) and second highest utilization of ED visits and hospital admissions in 2012.

**Table 2:** Healthcare utilization per patient and mortality rate in 2012

Variable	Mostly Healthy		Serious Acute Illness but Curable		Stable Chronic		Complex Chronic without Frequent Hospital Admissions		Complex Chronic with Frequent Hospital Admissions		End of Life		*p*-value
ED visits mean, (SD)	0.11	(0.38)	0.56	(0.95)	0.05	(0.31)	0.44	(0.94)	3.21	(3.16)	0.96	(1.36)	<0.001
Specialist Clinic visits mean, (SD)	2.42	(3.67)	8.20	(7.25)	2.04	(4.34)	8.52	(11.09)	24.30	(19.54)	27.48	(23.68)	<0.001
Hospital admissions mean, (SD)	0	(0)	1.18	(0.51)	0	(0)	0.44	(0.72)	4.33	(2.12)	1.50	(1.63)	<0.001
Mortality rate Number, (%)	666	(0.1)	266	(0.6)	286	(0.2)	2903	(3)	769	(16)	982	(33)	<0.001

Abbreviations: Emergency Department (ED). Numbers were presented as mean ± standard deviation or number (%) as appropriate

The prevalence of chronic diseases in each patient segment is presented in Table 3. The “Complex chronic with frequent hospital admissions” patients were significantly more likely to have more chronic diseases than the “Complex chronic without frequent hospital admissions” patients (3.5 ± 2.9, versus 2.4 ± 2.0; *p* < 0.001). Additionally, the top three common chronic diseases were Hypertension, Hyperlipidemia and Diabetes without chronic complication. Hypertension was the most common chronic disease, being present in 35% to 54% of patients with chronic diseases.

**Table 3:** Prevalence of chronic diseases in individual patient segments

Variable	Stable Chronic		Complex Chronic without frequent hospital admissions		Complex chronic with frequent hospital admissions		End of Life		*p*-value
	*N* = 177,550		*N* = 90,540		*N* = 4705		*N* = 2955		
Number of chronic diseases mean, (SD)	2.4	(1.3)	2.4	(2.0)	3.5	(2.9)	1.6	(1.6)	<0.001
Chronic Diseases^a^									
Diabetes without chronic complication (%)	30,823	(17.4)	23,506	(26.0)	1622	(34.5)	178	(6.0)	<0.001
Hypertension (%)	62,413	(35.2)	39,054	(43.1)	2540	(54.0)	433	(14.7)	<0.001
Chronic kidney disease without ESRF (%)	4954	(2.8)	6674	(7.4)	1341	(28.5)	42	(1.4)	<0.001
Asthma (%)	7041	(4.0)	3697	(4.1)	246	(5.2)	34	(1.2)	<0.001
Hyperlipidemia (%)	59,426	(33.5)	34,951	(38.6)	1885	(40.1)	219	(7.4)	<0.001
Osteoarthritis (%)	22,367	(12.6)	10,966	(12.1)	619	(13.2)	98	(3.3)	<0.001
Osteoporosis (%)	452	(0.3)	208	(0.2)	2	(0.0)	2	(0.1)	<0.001
Benign Prostatic hypertrophy (%)	1265	(0.7)	687	(0.8)	28	(0.6)	3	(0.1)	<0.001
COPD without cor pulmonale (%)	3084	(1.7)	4154	(4.6)	453	(9.6)	72	(2.4)	<0.001
Hyperthyroidism (%)	1720	(1.0)	422	(0.5)	14	(0.3)	2	(0.1)	<0.001
Hypothyroidism (%)	2915	(1.6)	1076	(1.2)	45	(1.0)	1	(0.0)	<0.001
Diabetes with chronic complications (%)	0	(0.0)	8935	(9.9)	450	(9.6)	12	(0.4)	<0.001
Stroke (%)	4737	(2.7)	8207	(9.1)	680	(14.5)	67	(2.3)	<0.001
CKD5 or ESRF (%)	950	(0.5)	3600	(4.0)	1241	(26.4)	40	(1.4)	<0.001
COPD with cor pulmonale (%)	2169	(1.2)	3763	(4.2)	441	(9.4)	72	(2.4)	<0.001
Major Depression	1302	(0.7)	5398	(6.0)	212	(4.5)	22	(0.7)	<0.001
Schizophrenia (%)	273	(0.2)	959	(1.1)	50	(1.1)	7	(0.2)	<0.001
Dementia (%)	331	(0.2)	671	(0.7)	65	(1.4)	4	(0.1)	<0.001
Bipolar disorder (%)	2	(0.0)	7	(0.0)	0	(0.0)	0	(0.0)	<0.001
Collagen Vascular diseases (%)	107	(0.1)	1412	(1.6)	128	(2.7)	15	(0.5)	<0.001
Anxiety (%)	693	(0.4)	2616	(2.9)	53	(1.1)	6	(0.2)	<0.001
Parkinson’s disease (%)	282	(0.2)	841	(0.9)	62	(1.3)	7	(0.2)	<0.001
Epilepsy (%)	289	(0.2)	1801	(2.0)	108	(2.3)	16	(0.5)	<0.001
Coronary heart disease, myocardial infarction	10,159	(5.7)	19,562	(21.6)	1513	(32.2)	122	(4.1)	<0.001
Atrial fibrillation (%)	0	(0.0)	718	(0.8)	91	(1.9)	2	(0.1)	<0.001
Hip fracture (%)	20	(0.0)	12	(0.0)	2	(0.0)	0	(0.0)	<0.001
Spine fracture (%)	185	(0.1)	827	(0.9)	77	(1.6)	8	(0.3)	<0.001
Moderate or severe liver disease, Liver cirrhosis	0	(0.0)	2843	(3.1)	245	(5.2)	34	(1.2)	<0.001
Any malignancy, non metastatic (%)	0	(0.0)	25,630	(28.3)	885	(18.8)	1915	(64.8)	<0.001
Thromboembolism: prosthetic valve, thrombosis, embolism (%)	0	(0.0)	42	(0.0)	12	(0.3)	3	(0.1)	<0.001
Pressure Ulcer (%)	10	(0.0)	166	(0.2)	29	(0.6)	2	(0.1)	<0.001
Heart failure & Fluid overload (%)	936	(0.5)	4880	(5.4)	811	(17.2)	40	(1.4)	<0.001
Peripheral vascular disease (%)	655	(0.4)	3223	(3.6)	372	(7.9)	25	(0.8)	<0.001
Metastatic disease (%)	6	(0.0)	181	(0.2)	16	(0.3)	1151	(39.0)	<0.001

^a^Based on ICD codes in the preceding 5 years. Abbreviations: end stage renal failure (ESRF), chronic obstructive pulmonary disease (COPD), KDIGO chronic kidney disease stage 5 (CKD-5). Numbers were presented as mean ± standard deviation or number (%) as appropriate

## Discussion

In this retrospective cross-sectional study of 825,874 eligible adult patients, we found that the majority of patients are healthy without chronic diseases. Although patients in the complex chronic disease with frequent hospital admissions segment represented 0.6% of the eligible population, this segment accounted for the highest hospital admissions and emergency attendances per patient, and second highest specialist clinic visits. Moreover, almost one in six patients deceased, suggesting that this is a high burden segment that requires further research into disease control, health behavior and most importantly, the bio psychosocial needs and coordination of care. Equally worth noting was the End of Life segment that accounted for highest specialist clinic visits despite having the highest mortality rate. These data provide support for the proof of concept that we can use a big data approach to segment the population into clinically meaningful groups with similar healthcare characteristics.

There are trade-offs between the simplicity and precision of segmentation. The Johns Hopkins Adjusted Clinical Groups System uses a granular system of diagnosis code mapping as the basis for different groupings [6]. Similarly, the 3 M Clinical Risk Groups system distributes patients among 272 groups for a more detailed risk analysis [7]. In this study, we had validated the feasibility of a practical expert-driven approach to segmentation as a first cut to categorize the patient population into six segments our RHS. There are several strengths of this approach. Firstly, this pragmatic simple categorization can be replicated to other RHSs in Singapore and worldwide that utilize the EHR, as the variables and healthcare utilization measures used in our study are commonly available. Similarly, it is possible to replicate our effort on a national level utilizing the National Electronic Health Record. Secondly, it is practically impossible to develop care models and intervention programs for each individual, and we aimed to segment into groups with largely similar characteristics to benefit from programs.

A key objective in population segmentation is to identify a population groups that are homogeneous enough in terms of healthcare needs or risk to enable rational customization of care. While our study managed to identify six distinct segments with different risk for healthcare utilization, it is possible that within each segment, patients may have different healthcare needs. Future work may add on to our study by conducting mixed method studies to assess the biopsychosocial needs of patients in each segment. This continuous iterative process allows for the refinement of the segmentation framework based on person-centered needs and inform the design of bundles of care that are sensitive and specific to patients’ needs. Nevertheless, in creating a regional health system population database, it allowed us to follow up patients in longitudinal cohort studies to monitor their disease progression and healthcare utilization trends, and predict for patients who are likely to transit from a low utilizer to a high utilizer segment for early intervention. Lafortune et al. [10] first segmented older persons into four homogenous categories of health status (i.e. health profiles) based on 17 indicators of prevalent health problems (chronic conditions; depression; cognition; functional and sensory limitations; instrumental, mobility and personal care disability). Latent transition analyses were then performed to study change in profile membership. Similarly, Casey JA et al. [25] highlighted the growing importance of electronic health records in epidemiologic investigations in population health research, through enhanced collection of social and behavior measures and linkage with vital records to develop longitudinal databases. Research ranging from cross-sectional studies within a given hospital, longitudinal studies on geographically distributed patients, environmental and social epidemiology, stigmatized conditions and predictive modeling can be performed to generate answers to key population health questions. We would be reporting on these trends and prediction models for our RHS in future works.

It is well established that metastatic disease is associated with disproportionate amount of healthcare spending. Nevertheless, patients with metastatic disease accounted for the highest specialist clinic attendances per patient despite their relatively shorter course of illness and high one-year mortality rate. Only a minority die at home in Singapore [19, 26] and the high consumption of hospital services at the end of life suggest that there is a role for home-based palliative care services and greater awareness of advance care planning (ACP). Ng et al. found that caregivers of palliative care patients had low awareness of terminal disease planning such as ACP and Advance Medical Directive [27]. There was a reticence to talk openly about issues surrounding end-of-life care as a result of cultural taboo and a fear that doing so will destroy hope. Since 2011, Singapore has implemented a national ACP program to place greater emphasis on developing ACP services among providers of public hospitals and the intermediate and long-term care. Community engagement, media advocacy and publicity also need to be in place to raise awareness and normalize conversations in care planning [28].

We believe that our study had proven the feasibility of using an EHR approach to segment the patient population into six non-overlapping segments using an adapted Ministry of Health Singapore segmentation framework. However, there are limitations to our study. First, variables in our dataset were restricted to those routinely collected in our EHR and administrative databases. As such, information about the functional status, caregiver availability and degree of social support were unavailable to refine the segmentation. We intend to overcome this in future by data linkage with researchers’ databases that are rich in social and behavioral data. Secondly, it is noteworthy that our database does not include patients who are exclusively managed in the private healthcare sector. Private sector coverage in Singapore is mainly for primary care and will predominantly impact the number and proportion of patients in the mainly healthy and serious acute illness but curable segments. Finally, our population database is unable to account for cross-utilization of healthcare services outside of the SingHealth RHS or out of hospital deaths. However, only hospital admissions were used to further stratify the patients with complex chronic diseases. Our results showed that the difference in hospital admissions between the complex chronic disease with or without frequent hospital admissions was almost 4, suggesting that cross utilization is unlikely to affect our conclusions.

## Conclusion

In this study, we described a practical segmentation framework and segmented the SingHealth RHS patient population into 6 distinct, non-overlapping patient segments. We found that patients in the complex chronic disease with frequent hospital admissions segment accounted for the highest hospital admissions and emergency attendances per patient and had a high mortality rate. This approach may be used as a model for further study to allow better understanding of population health and segmentation.

## Additional file

Additional file 1: Table S1. Population Segments in SingHealth Regional Health System, **Table S2.** Classification of Chronic Diseases. (DOCX 15 kb)

## Abbreviations

ACP: Advance care planning
ED: Emergency department
RHS: Regional health system
SGH: Singapore general hospital
SingHealth: Singapore health services
