# Supplementary material for: Evaluation of a practical expert defined approach to patient population segmentation: a case study in Singapore
Source: BMC Health Serv Res. 2017 Nov 23;17:771. doi: 10.1186/s12913-017-2736-8 (PMC5701430; doi:10.1186/s12913-017-2736-8)
Supplement: Additional file 1: Table S1. — Population Segments in SingHealth Regional Health System, Table S2. Classification of Chronic Diseases. (DOCX 15 kb) [file 12913_2017_2736_MOESM1_ESM.docx]

| **Additional file 1 Table S1: Population Segments in SingHealth Regional Health System** | | |
| --- | --- | --- |
| **Category** | **Definition** | **Descriptive Examples** |
| Mostly Healthy | No chronic diseases | A patient who saw the polyclinic for upper respiratory tract infection or attended the emergency department for gastritis. |
| Stable Chronic Disease | At least 1 chronic disease (that does not interfere with/restrict normal function or sufficient to trigger care seeking)** and 0 related admissions in one year | A patient who is seeing polyclinic for hypertension. |
| Serious acute illness but curable | No chronic diseases but had admissions for acute conditions e.g. acute appendicitis. Primary discharge diagnosis not related to defined chronic conditions in Table 2. | A patient who was admitted for acute appendicitis. |
| Complex Chronic without frequent hospital admissions | At least 1 chronic disease* (that interfere with/restrict normal function or sufficient to trigger care seeking)** and <3 related admissions in one year | A patient with hypertension, hyperlipidemia, atrial fibrillation, rheumatoid arthritis on follow up with hospital. |
| Complex Chronic with frequent hospital admissions | At least 1 chronic disease* (that interfere with/restrict normal function)** and recurrent admissions i.e. ≥3 in one year | A heart failure patient with frequent hospital admissions for fluid overload. |
| End of Life | Metastatic disease | A patient with metastatic cancer. |

* Full list of chronic diseases in supplementary table.

| **Additional file 1 Table S2: Classification of Chronic Diseases** |
| --- |
| **Stable Chronic Diseases** |
| 1. Diabetes without chronic complication |
| 2. Hypertension |
| 3. Chronic kidney disease without End Stage Renal Failure |
| 4. Asthma |
| 5. Hyperlipidemia |
| 6. Osteoarthritis |
| 7. Osteoporosis |
| 8. Benign Prostatic Hypertrophy |
| 9. Chronic Obstructive Pulmonary Disease without cor pulmonale |
| 10. Hyperthyroidism |
| 11. Hypothyroidism |
|  |
| **Complex Chronic Diseases*** |
| 1. Diabetes with chronic complications |
| 2. Cerebrovascular disease |
| 3. Chronic Kidney Disease Stage 5 or End Stage Renal Failure |
| 4. Chronic Obstructive Pulmonary Disease with cor pulmonale |
| 5. Major depression |
| 6. Schizophrenia |
| 7. Dementia |
| 8. Bipolar disorder |
| 9. Collagen Vascular diseases |
| 10. Anxiety |
| 11. Parkinson’s disease |
| 12. Epilepsy |
| 13. Coronary heart disease, myocardial infarction |
| 14. Atrial fibrillation |
| 15. Hip fracture |
| 16. Spine fracture |
| 17. Moderate or severe liver disease, Liver cirrhosis |
| 18. Any malignancy, non-metastatic |
| 19. Thromboembolism: prosthetic valve, thrombosis, embolism |
| 20. Pressure Ulcer |
| 21. Heart failure and Fluid overload |
| 22. Peripheral vascular disease |
|  |
| **End of life** |
| 1. Metastatic disease |

* A complex chronic disease is defined as one that that interfere with / restrict normal function or sufficient to trigger care seeking

**Appendix 1: Regional Health Systems in Singapore**

Taking a population health approach, the Ministry of Health Singapore has been transforming the care model from a hospital centric to a community centric model to avoid over-reliance on tertiary care. In 2011, the idea of regional health systems (RHSs) was proposed to integrate care across the care continuum, in particular vertical and horizontal integration of healthcare institutions and social care agencies within each region. Six RHSs were created, each being responsible to integrate care for a specific geographic region in Singapore. Each RHS is anchored by a tertiary hospital, supported by a community hospital providing intermediate and rehabilitation care and complete with linkages to primary care and long term care services in the region. In 2014, the Singapore Health Services (SingHealth) RHS was officially launched and consisted of primary to tertiary care institutions that account for care of nearly a million patients in Singapore. To integrate care effectively, it is crucial to have a deep understanding of the population health, characteristics and healthcare needs of patients served by the RHS. Therefore, population segmentation is a critical first step in population health management.
